# Experiences of clinical supervisors while supporting student veterinary nurses in the clinical learning environment

**DOI:** 10.1002/vetr.70178

**Published:** 2025-12-20

**Authors:** Susan L. Holt, Sarah Batt‐Williams, Sheena Warman

**Affiliations:** ^1^ Bristol Veterinary School University of Bristol Langford UK; ^2^ Centre for Veterinary Nursing Royal Veterinary College Hatfield UK

**Keywords:** clinical learning environment, clinical supervisor, communities of practice, situated learning theory, student veterinary nurse training, veterinary training practice

## Abstract

**Background:**

Clinical supervisors are responsible for student veterinary nurse training in veterinary practices. Clinical supervisors can have a significant impact on student experiences, so understanding how the role is perceived by all stakeholders is vital for the future development of veterinary nurse training.

**Methods:**

A qualitative, cross‐sectional design, using focus groups, was used to explore the perceptions of 16 clinical supervisors. Thematic analysis was used to identify themes across the focus group discussions. Situated learning theory was used to frame the study.

**Results:**

Clinical supervisors were motivated to provide a supportive learning experience for students and cited positive elements to this including supporting their own professional development. Challenges to supporting students included increasing student pastoral needs and a lack of protected time in practice.

**Limitations:**

The convenience recruitment strategy for this study may have led to volunteer (non‐response) bias, which could have reduced the trustworthiness of the findings.

**Conclusion:**

Robust support processes are important in training practices (TPs) and accredited education institutes (AEIs) to facilitate psychologically safe clinical environments, which support learning. It is beneficial if all members of the TP are involved in agreements with the AEI around the expectations of training students to ensure that time for training is appropriately managed and training expectations are clear.

## INTRODUCTION

In the UK, student veterinary nurses (SVNs) are required to complete a course provided by an RCVS accredited education institute (AEI) prior to consideration for professional registration with the RCVS. SVN training courses are required to uphold the RCVS Standards Framework for Veterinary Nurse Education and Training (the Framework).^1^ Within all of these courses, whether in higher education or further education, students are required to spend a minimum of 1800 hours within an AEI‐approved veterinary training practice (TP), undertaking clinical training and completion of the RCVS Day One Skills.^2^

The clinical learning environment is complex and dynamic; ensuring that students can navigate the inherent challenges requires AEI and TP organisational support, as reported for human nursing students.^3^ The AEI and TP leaders are responsible for continually evaluating training to ensure that learning objectives can be met that best prepare SVNs for practice.^4^ Failure to identify challenges and problems that are present in the clinical learning environment has been demonstrated to prevent effective learning and growth for human nursing students and can have negative impacts on SVNs.^5^, ^6^

The Framework Standard (6.6) states that a clinical supervisor must be assigned in the TP to support safe and effective learning; the AEI must ‘ensure all students are allocated a clinical supervisor responsible for confirming competency in the RCVS Day One Skills for veterinary nurses. All clinical supervisors must be either an RVN or MRCVS (UK practising), be experienced and able to demonstrate an experienced level of clinical skills and ongoing professional development’.^1^ The role of the clinical supervisor is referenced throughout the Framework guidance, pertinent points of which can be seen in Table 1. Adequate clinical supervisor support is reported to have a significant impact on SVN development and progress in the TP, so it is important to ensure this provision is well managed.^6^ The clinical supervisor is required to attend initial training and annual standardisation meetings with an AEI.

**TABLE 1:** RCVS Standards Framework guidance relating directly to the role of the clinical supervisor.^1^

Framework reference	Guidance relating to the standard
Introduction	… it is thus imperative that all requirements related to personnel involved in the training of student veterinary nurses includes consideration of clinical supervisors.
1.13	… clinical supervisors are well placed to support with developments for the practical assessments, in reflection of current practice.
4.1	Clinical supervisors may have undertaken coaching and mentoring training, or they may have exclusively attended training at the AEI for the specific programme they are supporting.
4.2	Clinical supervisors must be trained and annually standardised; their access to equality, diversity and inclusion training will likely occur during the clinical supervisor training sessions.
4.4	Clinical supervisors must also have the requisite time allowance, such as time afforded for teaching and assessment of the RCVS Day One Skills.
4.5	… individuals who regularly interact with students must also be able to facilitate supportive mechanisms. This is also true for clinical supervisors and is likely another area that can be covered during clinical supervisor training.
6.6	There is no requirement for clinical supervisors to have been registered for a specific length of time; however, it is expected that they themselves are suitably qualified and experienced to support the training of student veterinary nurses.
6.12	While all student veterinary nurses will be supported in the TP by a clinical supervisor, there will likely be times where the shift patterns do not align, or the clinical supervisor is on annual leave. Other members of the veterinary team can, in accordance with their skill and experience, assist with the training and assessment of the student veterinary nurse. This process must be clearly documented and auditable.

Abbreviations: AEI, accredited education institute; TP, training practice.

The requirement for 1800 hours work in a TP aligns with the theoretical framework of situated learning theory (SLT), with the student learning in an authentic environment where the learning will be applied.^7^ SLT cites learning through legitimate peripheral participation within an authentic setting among a community of practice, as an ‘apprentice’ with a ‘master’.^7^ In the context of SVN training, the clinical supervisor is the ‘master’.^7^, ^8^ The use of SLT, and the broader concepts of landscape of practice,^7^, ^8^, ^9^ provided a lens through which to design and conduct the research and analyse the presenting data in this non‐classroom learning situation, which supported an objective approach to problem solving to reduce the inherent researcher subjectivity and bias in qualitative research.^10^ Considering the veterinary landscape of practice,^8^ SVNs must navigate the landscape boundaries between the clinical workplace and AEI while also conforming to the RCVS requirements of professional practice and the professional relationships required with external partners (e.g., clients). When navigating these landscape boundaries, the student's professional identity will undergo regular change, which causes inherent challenges as they learn to understand their place within the profession.^8^ The clinical supervisor is well placed to act as a convenor as SVNs develop their professional identity across these boundaries, as this is where SVNs will require the most support, alongside support from the AEI.^8^, ^9^

### Current evidence in the veterinary profession

SVNs have reported that the supervisory relationship with their clinical supervisor has a significant impact on their training and progress in the TP.^6^, ^11^ Notable elements included the benefit of supportive and approachable clinical supervisors, working as positive role models with a broad level of clinical experience.^11^ In contrast, SVNs reported that limited time allocation for tutorial support and training, and poor engagement and lack of experience of the clinical supervisor were elements that detracted from their learning in the TP.^11^ These SVN perceptions were echoed in a second report that highlighted clinical supervisor support as vital for adequate Day One Skills progression and the development of professional identity.^6^ Research reporting the views of clinical supervisors has identified similar themes, with 67% of respondents in one study stating that they do not have time allocated in the TP to undertake their role.^11^, ^12^ A further concern raised by clinical supervisors was that of training, with over 47% of respondents in one report stating that they did not feel well prepared for their supervision journey.^13^ Psychological safety facilitates positive interpersonal and educational experiences for students supporting them to ask questions, share their ideas or speak up.^14^ While the Framework Standard (3.11) states that training must ensure that students ‘are protected from discrimination, harassment, incivility and other behaviours that undermine their performance or confidence’, it also highlighted, in the guidance notes, that recent research has shown that this requirement is not being effectively managed, particularly in clinical practice.^1^ Therefore, training of clinical supervisors in relation to creating psychological safety should be paramount for AEIs and could follow guidelines published for the human nursing field.^14^

Workforce shortage is reported to be a key challenge facing the veterinary nursing profession, and attrition is attributed to unsatisfactory pay, not feeling valued or rewarded and dissatisfaction with career opportunities.^15^ However, if appropriately resourced, the role of clinical supervisor can positively influence registered veterinary nurses’ (RVNs) job satisfaction and professional identity and support increased retention within the profession.^12^, ^16^, ^17^, ^18^ However, satisfaction and motivation will diminish if job stressors (e.g., the additional responsibilities of the clinical supervisor role) require sustained high effort with few opportunities to recover.^19^ The retention of experienced nurses in practice is important to ensure that the human resource of knowledge and experience is available to train SVNs during clinical learning experiences.^19^ Current research in the field of SVN training in the TP is limited and mostly conducted by the authors of the present study. However, research relating to student training in the human nursing field has been extensive and echoes the themes raised in veterinary nurse training.

### Human nursing literature

Veterinary nurse training draws many parallels with human nurse training. Notably, the RCVS Framework adopted the structure and format of the Standards of Proficiency for Registered Nurses, with permission from the Nursing and Midwifery Council.^1^ In human nursing, individuals supporting students in the workplace have titles other than clinical supervisor, but will be referred to here as mentors for clarity.^20^ Human nurse mentors have also reported that a lack of protected time was a challenge for their mentoring role in the workplace.^3^, ^21^, ^22^ Mentors play a vital role in creating a psychologically safe environment within the workplace, which facilitates training support from across the clinical team.^23^ Mentors have reported that they feel a lack of support from the wider clinical faculty, nursing managers and nursing educators when performing their mentor role, resonating with perceptions of clinical supervisors in veterinary practice.^6^, ^24^, ^25^, ^26^ The relationship and support from the AEI to the human nursing mentor has been cited as an area for improvement, with greater communication required to aid the understanding of the mentorship role and student requirements.^22^ A lack of formal training for the role was cited as a barrier and left human nursing mentors feeling inadequately prepared.^27^, ^28^ Positive factors for human nursing mentors have been reported to include development of leadership skills, support of reflective practice, keeping up to date, increased job satisfaction and watching students' progress.^3^, ^26^

### Study aims

The literature in the human and veterinary nursing fields has highlighted comparable challenges faced by those who undertake mentorship and supervisory roles.^3^, ^6^, ^11^, ^13^, ^15^, ^21^, ^26^, ^28^, ^29^ Understanding how clinical supervisors experience their role in supporting SVNs will be key to identifying and mitigating challenges to ensure that they can perform their role adequately with limited barriers and an adequate support network.

Previous research projects have explored the preparedness and perceptions of clinical supervisors using online surveys and explored SVN clinical training experiences via online surveys and interviews. The current study builds on previous research to further explore the clinical supervisor experience. The aim of the present study was to investigate the collective experiences of clinical supervisors in the TP when supporting SVNs. The research questions explored in the present study were as follows: How do clinical supervisors feel about their role? What benefits and challenges are inherent to the role? How can the experience of clinical supervisors be enhanced in the TP when supporting students?

## METHODS

The research team consisted of three female researchers. The first (lead) author is an RVN and academic with over 25 years’ clinical experience, including clinical supervision and over 9 years of working in academia, with a research masters and several publications within the same topic area. The second author is an RVN and academic who supported the data collection and analysis phase, and the third author is a professor of veterinary education and MRCVS with extensive experience in qualitative research in the field of veterinary education. As is standard in qualitative research, the lead author took a reflexive approach, acknowledging pre‐existing personal bias and preconceptions arising from their professional background. The impact of any bias or preconceptions was minimised through a robust research design, published theoretical frameworks and processes for systematic thematic analysis, and discussion of design, analysis and findings with co‐authors.

### Study design

A social constructivist epistemology guided the research design to fully appreciate the experiences of SVN clinical supervisors.^30^ Social constructivism is an educational theory that bases individual knowledge in socially mediated contexts, with the learner as an active participant in the learning process, rather than a passive receiver of knowledge.^31^ Therefore, a qualitative methodology was chosen to provide rich data, using focus groups as the data collection method. The focus group has five key characteristics that made it suitable for the current project: a small group of people, who possess certain defined characteristics, provide qualitative data, in a focused discussion, to help understand the topic of interest.^32^ Building on the social constructivist approach, SLT was chosen as an appropriate theoretical framework to guide the design and data analysis.^7^ Following detailed methodologies in qualitative research and being transparent throughout, including providing open access to the dataset, supports trustworthiness and credibility of the findings.^33^ The current project adhered to the consolidated criteria for reporting qualitative research (COREQ) guidelines for interviews and focus groups.^34^

The focus group structure followed the guidance by Krueger and Casey,^32^ starting with four focus groups with three to six participants in each. The key concepts analytical framework, described by Krueger and Casey,^32^ also informed the data collection plan (Table 2), which focused on identifying key concepts of importance, identifying a number of important ideas or experiences and then asking participants to validate these key concepts during the summary provided by the second author.

**TABLE 2:** Focus group plan.

Question type	Question
Opening: 2 minutes	1. Please introduce yourself and state how long you have been a clinical supervisor?
Introduction: 5‒10 minutes	2. What were the circumstances that led to you taking on this role?
Transition: 5‒10 minutes	3. Think back to when you started your role. What were your expectations of supporting students as a clinical supervisor?
Key concepts: 60 minutes	4. How confident do you feel when performing the various aspects of your role?
	5. What are the highs and lows of performing this role?
	6. What aspect of your clinical supervisor support do you feel is most valuable for your students?
	7. How do you feel your practice culture facilitates student learning
	8. Are there any other aspects of your role you would like to discuss that we may have missed in our questioning?
Ending: 5‒10 minutes—ask each participant in turn	9. Considering today's discussion, what key points do you feel are most important for enhancing clinical supervisor and/or student support in practice?
Summary: assistant facilitator to provide a summary—approximately 2 minutes of the content of the discussion	Following the summary, ask: How well does this capture what was discussed here today?

The focus group question guide was developed by the first and second authors. It was piloted with two clinical supervisors to obtain feedback, and no revisions were deemed necessary. The first focus group was initially conducted as a pilot session, but since no modifications were required, the data from this session were included in the final analysis. Data collection was planned to cease post priori, once theoretical saturation had been achieved (i.e., no new concepts were being generated and a consensus was achieved),^35^ following discussion by the first and second authors.

### Participant recruitment

Participant identification was achieved through convenience and snowball sampling. Clinical supervisors were required to be currently supporting UK SVNs in practice in the clinical supervisor capacity. Recruitment was achieved using an infographic posted on Facebook sites, including ‘VetNurseChatter’ and ‘ClinicalCoaches’, and distributed at the British Veterinary Nursing Congress and London Vet Show. These recruitment methods were selected due to the high numbers of target participants in their membership and attendance. AEIs were also asked to share the project with affiliated clinical supervisors.

The infographic contained a link to a Microsoft Form, providing the lead author with contact information from which further details and dates could be sent to prospective participants expressing an interest in the study. Consent was then obtained from those who indicated that they would like to attend a focus group after they had read the participant information sheet containing details of the researchers and reasons for conducting the research. No relationship was established with participants prior to the focus groups. There were 60 expressions of interest received, with 21 of these resulting in a commitment to attend a focus group. Five participants failed to attend the planned focus groups, without giving a reason, leaving 16 participants for the study.

### Data collection

The primary author facilitated the focus groups, with the second author present and providing the summary to participants at the end of the discussion. Focus groups were conducted online using Microsoft Teams (Microsoft Corporation). The online format was selected to facilitate the focus groups with geographically disparate participants with low cost and allowing participants to engage while maintaining personal boundaries, and it is considered to achieve comparable data collection when compared to in‐person discussions.^36^, ^37^ Focus groups were conducted between 6 November 2023 and 28 March 2024, with a total of 16 participants (between three and five participants in each group). Each focus group lasted between 50 and 71 minutes. There were no connection or call quality issues for researchers or participants during the interviews, and all participants had cameras on during the interview. The participants only took part in one focus group each. The primary and second authors and selected participants were present in each focus group, with no non‐participants present. Participants were made aware of who was conducting the research and why in the recruitment information and at the start of the focus groups. The second author made notes through each focus group for the purposes of a summary validation with each participant. Each focus group was small and homogenous in terms of demographics (in line with the profession demographics as reported in the 2024 RVN survey),^38^ which reduced some of the risks of focus group work, such as power imbalance and dominant speakers.^39^ The lead researcher also ensured that each participant was invited to speak within each area of the discussion to facilitate comparable air time. While the researchers acknowledge the limitations of online focus groups, such as reducing interaction between participants compared with face‐to‐face facilitation, the convenience to participants was considered to outweigh the limitations.^40^

### Data analysis

The video recording function was used on the platform, autogenerating a verbatim transcription that was checked, validated and converted into intelligent verbatim by the second author using the video recordings.^41^ The recordings and transcripts were stored on a password‐protected Microsoft SharePoint site. The second author also added non‐verbal communication noted during the focus groups, such as nodding by other participants.^41^ All names were removed to protect the anonymity of individuals, practices and AEIs. The transcripts were analysed by the first and second authors using the theoretical thematic analysis (TTA) method, as discussed by Braun and Clarke.^42^ TTA is guided by an existing theory, in this case SLT, as well as by the researcher's standpoint, disciplinary knowledge and epistemology. The analysis here followed the five steps described in detail by Castleberry and Nolan,^43^ including compiling, disassembling, reassembling, interpreting and concluding the data. For the final three stages, the online platform Mural was used to organise the codes and themes from across all the focus group data. The analysis stages were completed after each focus group, and theoretical saturation was achieved after four focus groups, with no new concepts being presented.

## RESULTS

**FIGURE 1:**
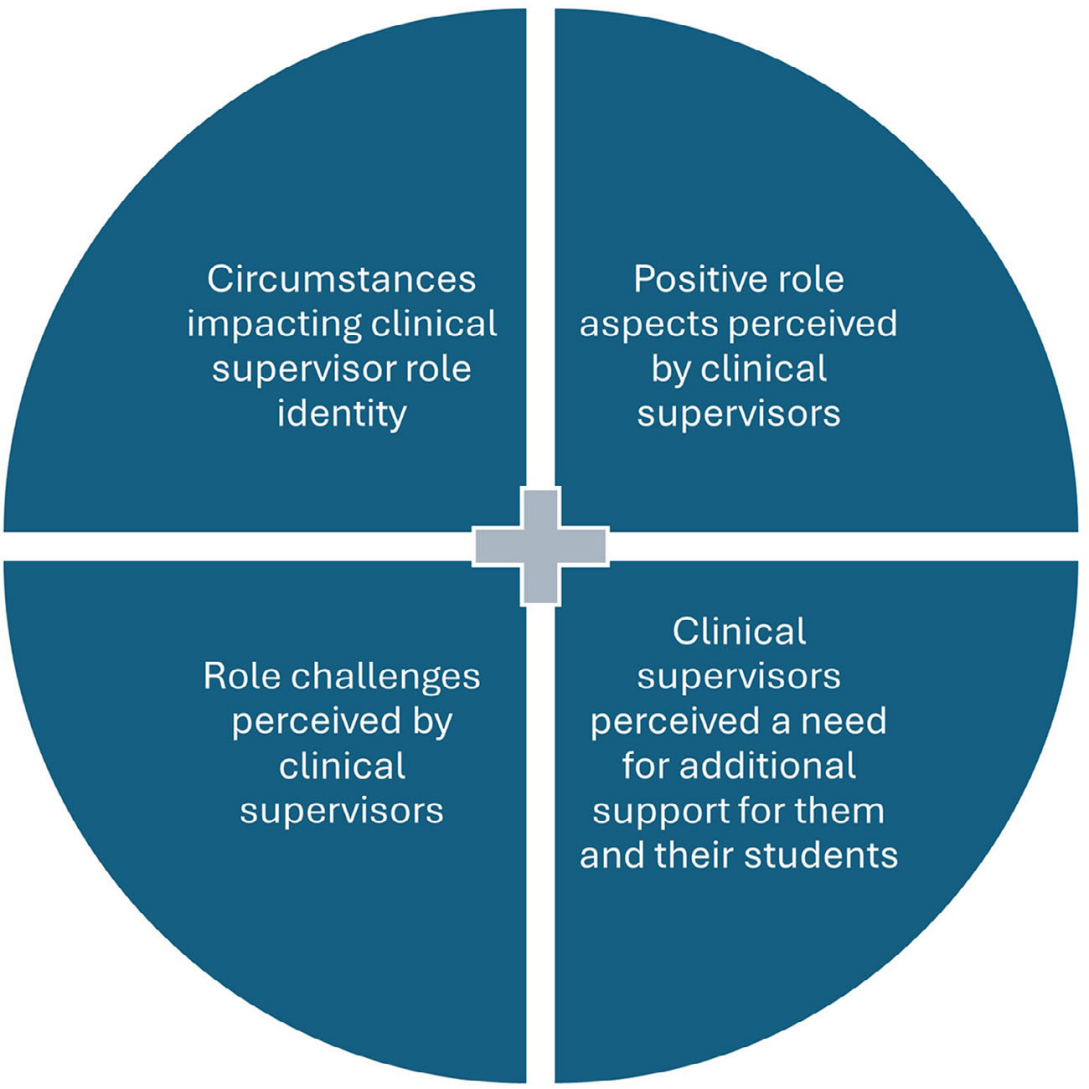
Infographic showing the main themes identified following analysis of the focus group data.

All 16 participants were RVNs and had been acting as clinical supervisors for at least 2 years. Following the data analysis, four themes were identified, with two to three sub‐themes within each (Figure 1). Each participant was given a unique number (e.g., P1) to maintain anonymity, which was used when quoting for theme validation.

### Themes

#### Circumstances impacting clinical supervisor role identity

This theme covered the clinical supervisors’ opinions on what informed their sense of identity when performing the role of clinical supervisor and was divided into the following three sub‐themes: personal experience as a student of clinical supervision enhanced motivation; non‐supervisor roles and experience impact confidence for student supervision; and role responsibilities beyond the teaching of clinical skills are well understood.

(1) Personal experience as a student of clinical supervision‐enhanced motivation.

The participants discussed why they had entered the role, with their choice being linked to their own experience as a student. Ensuring  students had a good experience in their training was a strong motivator for taking on the role; motivation was linked not only to their own positive experience but also to those who had a negative student experience, wanting to improve experiences for students under their supervision.

I remember, having quite good clinical coaches and yeah, it's just kind of giving other students that opportunity. P10

… I had felt when I was training myself, I sometimes had some coaches or mentors that didn't particularly want to be doing it. But we're sort of forced to do it in practice, and I felt that that affected my training, and I never wanted anyone to be in that situation themselves …. P11

(2) Non‐supervisor roles and experience can impact confidence for student supervision.

There were discussions around how confident the clinical supervisors felt in performing the role, which was linked to the other roles they were performing in practice.

I'm more a wards like medical person rather than surgical, so all of the anaesthesia and that stuff I pass on to people […] who have more knowledge and are more passionate about that side of things. P7

I definitely found that doing line management as well has helped with that mentoring side of things and definitely those skills marry up really nicely. P12

(3) Role responsibilities beyond the teaching of clinical skills are well understood.

The participants showed a clear understanding of how their role might support students in practice beyond teaching and assessing their clinical skills. Being an advocate, creating space for learning experiences and providing pastoral support were valued. The aspect of allowing the students time in practice for tutorials was also discussed as an important requirement, alongside tailoring support for individual needs. Being open and honest with students about personal skills and experience was also raised as important.

… tutorials, practical teaching, and I guess sort of pastoral support as well … So, I just wanted to make sure that I was, you know, there to support them should they need any of that pastoral support. P16

… I think it's probably having someone that is a true advocate for them in a busy environment. P1

#### Positive role aspects perceived by clinical supervisors

Within this theme, two subthemes emerged related to the positive aspects of performing the role in clinical practice.

(1) Watching students’ progression is a rewarding aspect of the role.

A resounding positive aspect for participants was watching their students’ progress with their skills and confidence and completing their training.

… seeing them grow in confidence with that is really nice and also seeing them, you know, sort of happily do it next time and not be too standoffish with it. P13

(2) Enhancing continued professional development is a personal benefit of clinical supervision.

The participants highlighted that performing the role helped them keep up to date and develop professional skills.

it's [clinical supervising] helped me to progress with my teaching qualification as well P4

… it's [clinical supervising] always trying to make sure that you […] are up to date. P2

#### Role challenges perceived by clinical supervisors

There were challenges for participants when performing the role of a clinical supervisor, and these were divided into three sub‐themes.

(1) Supporting struggling students can be challenging and time consuming.

The participants discussed that disengaged or less proactive students posed challenges in managing timely progression, and for some clinical supervisors, a feeling of helplessness and disappointment arose when trying to manage appropriate support. The participants also expressed a sense that students are requiring more support in recent years, particularly with maintaining positive mental health, and this was very time consuming to provide.

… it can be quite tricky trying to assist them with getting to that point when they are, maybe, you know, not being the most organised or not making use of the help and resources available to them, that can be slightly frustrating. P16

when you see them struggling and you don't feel that you can help them all … P4

(2) Inconsistencies and poor communication with the AEI are detrimental to the clinical supervisor‒AEI relationship.

These challenges are related to the support from the AEI and the varying course requirements, in addition to the range of online platforms used by institutions. The logging of the Day One Skills platforms varies across AEIs and have more recently been in flux. Clinical supervisors were having problems navigating these platforms, with a perceived lack of standardised guidance coming from the AEIs. The participants also felt that AEIs were not always encouraging a collaborative relationship with the practice team, which hindered access to support and created feelings of despondence and frustration.

… I think one of the big lows is going back to Participant 8's point is the communication back from college, because every […] 3 months, they would change the goal posts. [lots of nodding in agreement from other participants] … it [online logging platform] would just keep coming back and just kept coming back because they weren't communicating properly, and I think that was one of our biggest lows …. P9

I'm not that good at communicating with the providers because that can sometimes be quite a negative process …. P8

(3) Under resourcing in practice impacts the quality of clinical supervisor and student support.

The final sub‐theme here related to challenges that originated within the TP. Challenges included poor time management and not providing time for the clinical supervisor and student to support the training needs. Lack of training time was linked to staffing shortages in some circumstances. Staff shortages also led to a knock‐on effect of treating students such as qualified staff, with a level of expectation placed on students beyond their level of competence.

… can be challenging in terms of staffing to get the students their time that they need, they deserve, they should have and so I think, yeah, we need to be better at kind of facilitating that for them. P6

I think that they should be employed as an extra, but in fact they're just pushed into […] working outside their competency quite early on … P8

#### Clinical supervisors perceived a need for additional support for them and their students

Within this theme, the participants discussed the ways they felt support could be improved for them and their students and these were divided into two sub‐themes.

(1) A supportive training culture, including from non‐clinical management, is essential for a good clinical supervisor and student experience.

Having a supportive team culture, including from managerial staff, was important for supporting students and clinical supervisors, with a need for the whole team to be aware of the requirements of training SVNs. Another key request was having protected time to undertake training.

… practice just expect a little bit too much from students and a little bit too much from me as clinical supervisor. … our [the AEI] asked the actual training practice principal now to be part of the meeting …. P5

(2) Comprehensive clinical supervisor training and support from the AEI is a firm desire.

There was discussion around the level of training and support that is provided by AEIs for clinical supervisors and how this could be improved. Providing more detailed training prior to starting the role was deemed important, with the inclusion of pastoral support. Greater standardisation between AEI training and requirements for each student was also deemed necessary, and perhaps long overdue.

… it's reached a stage where clinical supervisors should have some more formal training rather than just this sort of standardisation. Just a bit more about giving emotional support, wellbeing, safeguarding … P2

How it [online logging platform] works and what is expected, because like you say, you can get 2‒3 months down the line and it [online logging platform] gets verified and then put back at you. P12

## DISCUSSION

To address the research questions, the present study explored the shared experiences of 16 clinical supervisors, gathered through four focus groups, regarding their support of SVNs during a TP. SLT was used to frame the study. While there were positive aspects to undertaking the role, such as continued professional development, there were shared challenges, such as lack of time, which require a holistic approach to improve management. Clinical supervisors desired additional support to enhance their role and improve their student experience.

### How do clinical supervisors feel about their role?

RVNs had experiences related to their own training that had impacted their motivation to engage in a clinical supervisor role. Whether they had a positive or negative experience, the participants felt that they wanted to provide positive support to their students. Such motivations may be an effect of volunteer bias, with those motivated to participate also being motivated to perform their role to a high standard.^44^ The negative experiences that some reported from their own clinical supervision during training echoes comments reported by SVNs that the supervisor and student relationship is not always as the student would desire.^6^, ^11^ Empowering and encouraging students to report to their AEI when relationships in the practice are challenging could instigate early intervention and support for the TP and SVN, thus improving the student experience. There is a wide range of topics for teaching and learning in veterinary nursing and the clinical supervisors support all these areas in the TP. However, as the Framework guidance (6.12) states, other staff can also assist. It is clear from the results that clinical supervisors can refer some aspects of training to more experienced team members for specific topic areas such as anaesthesia or radiography. Doing so will enhance student training as they will have access to the wider community of practice within the TP, which SLT frames as vital for learning and development and helps build professional identity.^45^ While the Framework requirement (6.6) states there is no minimum time that professionals should be qualified to perform the clinical supervisor role, it is clear from the results that additional experiences can enhance confidence and performance of the role, which will in turn benefit the student. Therefore, experience should be considered when selecting staff for the supervisor role, alongside additional responsibilities undertaken, which could limit capacity for student support. In the current workforce shortage, this may be challenging. There are references to pastoral support from the clinical supervisor for the student in the Framework, including (4.2), relating to equality, diversity and inclusivity (EDI), and (4.5), relating to supportive mechanisms (Table 1). The results showed that clinical supervisors were certainly aware of requirements relating to EDI and pastoral care; again, volunteer bias may have impacted these results, and this may not be representative of the population.

### What benefits and challenges are inherent to the role?

Veterinary nurses who have opportunity for career progression are reported as 71% less likely to leave the profession, and those who report job satisfaction are 85% more likely to stay in the profession.^46^ Clinical supervisors cited positive elements, which would increase job satisfaction and could enhance career progression when performing the role of clinical supervisor, such as watching student progress and enhancing their own professional development. Retaining professionally experienced clinical supervisors is important as such experience will have positive impacts on student satisfaction in the TP.^6^, ^11^ Highlighting these benefits might help motivate staff to consider the role positively. A sense that the role is valued by the TP is important; this might be achieved through, for example, increased clinical supervisor remuneration and greater professional status in the TP.^12^, ^47^, ^48^ Currently, in higher education veterinary nursing programmes, the government states that clinical placement as part of a degree course, if less than 1 year, is exempt from minimum wage.^49^ Students studying further education courses will be employed and paid by the TP. Currently, the authors are not aware of any higher education institutes that pay the TP to place SVNs. Therefore, the cost‒benefit of SVN training to practice could be difficult to quantify, especially if the TPs are expected to pay more to clinical supervisors.

Reviewing the payment structures involved with SVN training might be an appropriate discussion for all stakeholders. Discussions could include a consideration of SVN remuneration to support the cost of living challenges reported in students,^50^ as well as payment from the AEI to the practice, or more support from the AEI in managing pastoral needs. Reducing the pastoral responsibility of the clinical supervisor could lessen the time and emotional burden of supporting students. However, it is appreciated that these financial matters are challenging elements to manage and would require careful consideration by all stakeholders.

Challenges perceived by clinical supervisors when performing the role are congruent with challenges reported by SVNs, including a lack of time for training and tutorials, lack of defining and managing expectations of both students and supervisors, lack of protecting the role of students compared to qualified staff and meeting student pastoral needs.^6^, ^11^ Clinical supervisors reported that while they understood they had a responsibility for student pastoral support, they were not afforded the time in practice to do this effectively, which led to frustration. These findings are all misaligned with the Framework requirements and demonstrate that while the Framework exists, its requirements may not always be clearly met by the TP, and the AEI is responsible for monitoring and addressing such shortfalls. These challenges are similar to those reported by human nurse mentors, for whom insufficient support can result in reduced motivation to fulfil their mentoring role.^51^

Clinical supervisors also noted a perceived increase in the students’ need for pastoral support, particularly around mental health. Providing pastoral care created a feeling of helplessness and frustration for supervisors when they did not feel they could provide the required support. This finding also matches the reports of wellbeing and mental health challenges by SVNs.^11^ Mental health disorders are reported to be highly prevalent and increasing in young adults, with a higher occurrence in females, which can impact educational attainment and occupational function.^52^, ^53^ Current SVN enrolment figures show a total population of 6348, with 6038 (95.1%) identifying as females and 3824 (60.2%) being aged 25 years or under (Hedges V, RCVS, personal communication, 7 November 2024). Therefore, a significant proportion of SVNs match the demographic of the reported increased incidence of mental health disorders.^52^, ^53^

According to Maslow's hierarchy of needs, students cannot achieve effective learning or reach self‐actualisation if their fundamental needs, such as safety and a sense of social belonging, are not met.^54^ All those supporting students will benefit from clear processes to enable appropriate responses to the symptoms of poor mental health and wellbeing. As articulated in SLT, learning involves a process of personal transformation; to learn is, in essence, to become a different person.^45^ For this transformation to be positive and meaningful in the professional context, a psychologically safe workplace is required.^55^ Creating a psychologically safe space allows for positive collaboration, voicing ideas, experimentation, development and a ‘no blame’ culture and is critical in reducing errors and enhancing safety in healthcare settings.^56^ Creating a psychologically safe training environment works best with investment from the whole TP team and support from the AEI.^55^, ^57^

The AEI was perceived by clinical supervisors as providing inconsistent advice regarding the completion of online logging platforms. Given the lack of time for support and training needs reported within the TP, having to go over aspects of the progress log again due to unclear expectations or negative feedback was challenging and was perceived as undermining the relationship between the AEI and clinical supervisors. It is highly beneficial if the AEI and TP work in close collaboration, with both stakeholders able to initiate new ideas, freely explore alternatives and share insights without undue domination from any party.^8^ Each student is also a stakeholder in this relationship. Strong partnerships will allow all stakeholders to become “system convenors”, fostering social learning between landscapes, as described by Wenger‐Trayner et al.^8^

### How can the experience of clinical supervisors be enhanced in the TP when supporting students?

When discussing practice support, clinical supervisors felt that there was a need for more time protected for them and their students to undertake training and support activities, in line with the Framework (3.7, 4.4) requirement. AEIs are responsible for ensuring that this takes place with regular student and TP contact, carefully reviewing rota records and monitoring progress. It is clear from clinical supervisors and students that this is not always achieved as desired.^6^, ^11^ There was also a report that students are being put under undue pressure and treated as qualified staff, which could impact their professional identity and confidence and does not align with the Framework (3.7) requirements. Students who experience repeated failure when placed in situations they are not ready for will experience decreased self‐esteem and increased stress,^58^ which may in turn negatively impact their ability for transformative learning. Furthermore, this is also contrary to the RCVS Code of Professional Conduct, placing all involved in inappropriate delegation at risk of punitive action. AEIs should take this into account when determining how frequently to review a TP status, to ensure that risks to training are managed promptly. Additionally, whistleblowing policies should be clearly communicated to students, alongside efforts to empower them to report concerns confidently.

To address challenges related to the AEI's online logging platform requirements, AEIs could engage TPs as key stakeholders in developing these requirements while ensuring alignment with the RCVS Framework. Such collaboration would promote joint ownership, clearer expectations and stronger commitment from both parties. Feedback to the TP and clinical supervisor is best if void of power dynamics, to remove a feeling of ‘us and them’ from the relationship. All parties are working to support SVN training, so creating a collaborative partnership will be beneficial for all parties. Clinical supervisors cited that from the onset of a training relationship, it is beneficial if the AEI includes all members of the TP in the required expectations of the training agreement. Agreement is particularly important for the senior leadership team, who will be responsible for managing staffing levels, workloads and rotas. Students have also cited that a lack of support from management can have a negative impact on their training, echoing the perceptions of the clinical supervisors.^6^ Involving all parties at the outset of the training relationship can aim to provide clear expectations for the TP and may improve time allocation for training needs.

Clinical supervisors also stated that they required more comprehensive training both initially and annually in standardisation meetings. Currently, beyond the Framework requirements, there is no specific training syllabus for clinical supervisors, and training is left to the interpretation of each individual AEI. The Framework requirements (4.2 guidance) state that access to EDI training will likely occur during the clinical supervisor training sessions. The word likely, used here, could be considered insufficient with respect to EDI training for clinical supervisors, although it is vital for adequate student training and support. As discussed, mental health support needs are growing among the SVN population demographic, and clinical supervisors have now cited these as challenges in clinical training.^6^, ^11^, ^12^, ^13^ Therefore, perhaps it is time to review this element of training for clinical supervisors and create more formal processes to ensure that they are equipped and supported by the AEI for the demands of their role.

### Limitations

The convenience recruitment strategy must be considered when interpreting the results, as volunteer (non‐response) bias is a reported effect in research.^59^ The volunteer effect could reduce credibility and trustworthiness in qualitative research, such as seen in health habit studies.^44^ Research has demonstrated that forcing participation does not necessarily improve credibility.^60^ However, the authors recognise that this will have impacted some aspects of the results, such as motivations for undertaking the role.

## CONCLUSION

The clinical supervisors in this study demonstrated a strong commitment to all aspects of student support. Although they identified positive elements within their role, they also encountered several challenges that led to feelings of frustration. It is important that the AEI and TP work to foster positive collaboration in making decisions about student support provision, with a focus on appropriate time management for training needs and clear requirements for online logging platforms. Considering the growing number of challenges around pastoral support and maintaining mental health and wellbeing in SVNs, the authors suggest a holistic review of this provision to develop more robust processes. TPs, AEIs and the Framework guidance should support psychologically safe clinical environments. Active consideration and consistency in how clinical supervisors are resourced, trained and supported should aim to positively impact the supervisor and student experience.

## AUTHOR CONTRIBUTIONS

*Project idea and design lead, recruitment activities including social media posts, personal contacts, and British Veterinary Nursing Congress. Focus group facilitator and main author for the data. Responsible for final submission and ensuing amendments*: Susan L. Holt. *Critical support during design phase. Support with recruitment activities at London Vet Show and via contacts and social media. Present during all focus groups to provide participants with a summary of the discussion for agreement. Transcriptions of all focus group data, data analysis with primary author, critical review of write‐up prior to submission*: Sarah Batt‐Williams. *Project supervisor for design support and guidance at all phases and critical review of final write‐up prior to submission*: Sheena Warman.

## CONFLICT OF INTEREST STATEMENT

The authors declare they have no conflicts of interest.

## FUNDING INFORMATION

The authors received no specific funding for this work.

## ETHICS STATEMENT

Ethical approval was received from the University of Bristol Faculty of Health Sciences Research Ethics Committee (code 15068).

## Data Availability

Due to the sensitivity of the data involved, these data are published as a restricted dataset at the University of Bristol Research Data Repository data.bris, at https://doi.org/10.5523/bris.rbxo49osrpcq26nun4tvilwfv. The metadata record published openly by the repository at this location clearly states how data can be accessed by bona fide researchers. Requests for access will be considered by the University of Bristol Research Data Service, which will assess the motives of potential data re‐users before deciding to grant access to the data. No authentic request for access will be refused and re‐users will not be charged for any part of this process.

## References

[1] Royal College of Veterinary Surgeons . RCVS standards framework for veterinary nurse education and training. 2024 [cited 2025 Jan 4]. Available from: https://www.rcvs.org.uk/setting‐standards/accrediting‐primary‐qualifications/accrediting‐veterinary‐nursing‐qualifications/rcvs‐standards‐framework‐for‐veterinary‐nurse‐education‐and/

[2] Royal College of Veterinary Surgeons . Record of training. 2018 [cited 2020 Sept 7]. Available from: https://www.rcvs.org.uk/document‐library/record‐of‐veterinary‐nurse‐training/

[3] Kakyo TA , Xiao LD , Chamberlain D . Benefits and challenges for hospital nurses engaged in formal mentoring programs: a systematic integrated review. Int Nurs Rev. 2022;69(2):229–238. 34820833 10.1111/inr.12730

[4] Flott EA , Linden L . The clinical learning environment in nursing education: a concept analysis. J Adv Nurs. 2016;72(3):501–513. 26648579 10.1111/jan.12861

[5] Jamshidi N , Molazem Z , Sharif F , Torabizadeh C , Najafi Kalyani M . The challenges of nursing students in the clinical learning environment: a qualitative study. Sci World J. 2016;2016:1846178. 10.1155/2016/1846178 PMC4913003 27366787

[6] Holt SL , Mason J , Farrell M , Corrigan RH , Warman S . Exploring the sociocultural experiences of student veterinary nurses in the clinical learning environment through the lens of situated learning theory. Vet Rec. 2024;194:e3956. 38468387 10.1002/vetr.3956

[7] Lave J , Wenger E . Situated learning: legitimate peripheral participation. Cambridge University Press; 1991.

[8] Wenger‐Trayner E , Fenton‐O'Creevy M , Hutchinson S , Kubiak C , Wenger‐Trayner B . Learning in landscapes of practice: boundaries, identity, and knowledgeability in practice‐based learning. Routledge; 2014.

[9] Wenger E . Communities of practice: learning, meaning, and identity. Cambridge University Press; 1998.

[10] O'Brien BC , Battista A . Situated learning theory in health professions education research: a scoping review. Adv Health Sci Educ Theory Pract. 2020;25(2):483–509. 31230163 10.1007/s10459-019-09900-w

[11] Holt SL , Farrell M , Corrigan RH . Veterinary nursing students’ experience in the clinical learning environment and factors affecting their perception. J Vet Med Educ. 2024;51(3):357‒368. 37083602 10.3138/jvme-2022-0133

[12] Batt‐Williams, S . Yon, E . Investigation into the experiences of clinical supervisors and their perceptions of their role, in addition to the factors that affect them—Part 3. Vet Nurs J. 2022;36:49–54.

[13] Holt SL , Vivian SR , Brown H . Training and preparedness of clinical coaches for their role in training student veterinary nurses in the United Kingdom: an exploratory inquiry. J Vet Med Educ. 2021;49(1)e20200100. 10.3138/jvme-2020-0100 33657339

[14] Hardie P , O'Donovan R , Jarvis S , Redmond C . Key tips to providing a psychologically safe learning environment in the clinical setting. BMC Med Educ. 2022;22(1):816. 36443730 10.1186/s12909-022-03892-9 PMC9706932

[15] Royal College of Veterinary Surgeons . Recruitment, retention and return in the veterinary nursing profession. [cited 2024 May 25]. Available from: https://www.rcvs.org.uk/news‐and‐views/publications/recruitment‐retention‐and‐return‐in‐the‐veterinary‐nursing/

[16] Morrison TL , Brennaman L . What do nursing students contribute to clinical practice? The perceptions of working nurses. Appl Nurs Res. 2016;32:30–35. 27969047 10.1016/j.apnr.2016.03.009

[17] Vivian SR , Holt SL , Williams J . What factors influence the perceptions of job satisfaction in registered veterinary nurses currently working in veterinary practice in the United Kingdom? J Vet Med Educ. 2022;49(2):249–259. 34156909 10.3138/jvme.2020-0119

[18] Page‐Jones S , Abbey G . Career identity in the veterinary profession. Vet Rec. 2015;176(17):433. 25564471 10.1136/vr.102784

[19] Coates CR . Motivation and job satisfaction in veterinary nursing. Vet Nurse. 2015;6(6):360–365.

[20] Sibson L . Clinical coaching for veterinary nurses–supporting students in practice. Vet Nurs J. 2011;26(5):168–175.

[21] McIntosh A , Gidman J , Smith D . Mentors’ perceptions and experiences of supporting student nurses in practice. Int J Nurs Pract. 2014;20(4):360–365. 25157940 10.1111/ijn.12163

[22] Broadbent M , Moxham L , Sander T , Walker S , Dwyer T . Supporting Bachelor of Nursing students within the clinical environment: perspectives of preceptors. Nurse Educ. 2014;14(4):403–409. 10.1016/j.nepr.2013.12.003 24439528

[23] Hilli Y , Melender HL , Salmu M , Jonsén E . Being a preceptor—a Nordic qualitative study. Nurse Educ Today. 2014;34(12):1420–1424. 24801746 10.1016/j.nedt.2014.04.013

[24] Kalischuk RG , Vandenberg H , Awosoga O . Nursing preceptors speak out: an empirical study. J Prof Nurs. 2013;29(1):30–38.

[25] Kurniawan MH , Bahtiar B . Nurse preceptor experience in preceptorship program: a systematic literature review of qualitative studies. Int J Nurs Health Serv. 2018;1(1):35–48.

[26] Rylance R , Barrett J , Sixsmith P , Ward D . Student nurse mentoring: an evaluative study of the mentor's perspective. Br J Nurs. 2017;26(7):405–409. 28410033 10.12968/bjon.2017.26.7.405

[27] Ryan C , McAllister M . The experiences of clinical facilitators working with nursing students in Australia: an interpretive description. Collegian. 2019;26(2):281–287.

[28] Panzavecchia L , Pearce R . Are preceptors adequately prepared for their role in supporting newly qualified staff? Nurse Educ Today. 2014;34(7):1119–1124. 24679925 10.1016/j.nedt.2014.03.001

[29] Batt‐Williams S . Yon E . Investigation into the experiences of clinical supervisors and their perceptions of their role, in addition to the factors that affect them—Part 2. Vet Nurs J. 2022;36:44–48.

[30] Burr V . Social constructionism. Routledge; 2015.

[31] Thomas A , Menon A , Boruff J , Rodriguez AM , Ahmed S . Applications of social constructivist learning theories in knowledge translation for healthcare professionals: a scoping review. Implement Sci. 2014;9(1):54. 24885925 10.1186/1748-5908-9-54 PMC4040365

[32] Krueger RA , Casey MA . Focus Groups: A Practical Guide for Applied Research. SAGE Publications; 2014.

[33] Ahmed SK . The pillars of trustworthiness in qualitative research. J Med Surg Public Health. 2024;2:100051.

[34] Tong A , Sainsbury P , Craig J . Consolidated criteria for reporting qualitative research (COREQ): a 32‐item checklist for interviews and focus groups. Int J Qual Health Care. 2007;19(6):349–357. 17872937 10.1093/intqhc/mzm042

[35] Saunders B , Sim J , Kingstone T , Baker S , Waterfield J , Bartlam B , et al. Saturation in qualitative research: exploring its conceptualization and operationalization. Qual Quant. 2017;52(4):1893. 29937585 10.1007/s11135-017-0574-8 PMC5993836

[36] Gray LM , Wong‐Wylie G , Rempel GR , Cook K . Expanding qualitative research interviewing strategies: Zoom video communications. Qual Rep. 2020;25(5):1292‒1301.

[37] Hanna P . Using internet technologies (such as Skype) as a research medium: a research note. Qual Res. 2012;12(2):239–242.

[38] Campbell B , Plowden C , Robinson D . The 2024 survey of the veterinary nursing profession. RCVS; 2024 [cited 2025 Sept 5]. Available from: https://www.rcvs.org.uk/news‐and‐views/publications/the‐2024‐survey‐of‐the‐veterinary‐nursing‐profession‐report/

[39] Rutledge SA , Gilliam E , Closson‐Pitts B . ‘I'm being heard right now’: amplifying individual voice through scaffolded focus groups. Int J Soc Res Methodol. 2023;26(1):67–82.

[40] Jones JE , Jones LL , Calvert MJ , Damery SL , Mathers JM . A literature review of studies that have compared the use of face‐to‐face and online focus groups. Int J Qual Methods. 2022;21:16094069221142406

[41] Upwork . Transcribing examples: different types of transcription explained. [cited 2024 Oct 20]. Available from: https://www.upwork.com/resources/types‐of‐transcriptions

[42] Clarke V , Braun V . Successful qualitative research: a practical guide for beginners. Sage Publication; 2013.

[43] Castleberry A , Nolen A . Thematic analysis of qualitative research data: is it as easy as it sounds? Curr Pharm Teach Learn. 2018;10(6):807–815. 30025784 10.1016/j.cptl.2018.03.019

[44] Hegedus EJ , Moody J . Clinimetrics corner: the many faces of selection bias. J Man Manip Ther. 2010;18(2):69–73. 21655388 10.1179/106698110X12640740712699 PMC3101070

[45] Lave J , Wenger E . Situated learning: legitimate peripheral participation. Cambridge University Press; 1991.

[46] Jeffery A , Taylor E . Veterinary nursing in the United Kingdom: identifying the factors that influence retention within the profession. Front Vet Sci. 2022;9:927499. 36504871 10.3389/fvets.2022.927499 PMC9728522

[47] Batt‐Williams, S . Yon, E . Investigation into the experiences of clinical supervisors and their perceptions of their role, in addition to the factors that affect them—part 1. Vet Nurs J. 2022;36:38–43.

[48] Batt‐Williams S , Yon E . Investigation into the experiences of clinical supervisors and their perceptions of their role, in addition to the factors that affect them—Part 1. Vet Nurs J. 2022;36:38–43.

[49] Department of Business and Trade . Eligibility for minimum wage. 2024 [cited 2025 Jan 4]. Available from: https://www.gov.uk/guidance/calculating‐the‐minimum‐wage/eligibility‐for‐the‐minimum‐wage#:~:text=The%20main%20exemptions%20which%20mean,does%20not%20exceed%201%20year

[50] Dabrowski V , Atas N , Ramsey T , Howarth N . ‘Money anxiety’: understanding HE students' experiences of the cost‐of‐living crisis. Soc Policy Adm. 2025;59(2):280–292

[51] Benny J , Porter JE , Joseph B . A systematic review of preceptor's experience in supervising undergraduate nursing students: lessons learned for mental health nursing. Nurs Open. 2023;10(4):2003–2014. 36336826 10.1002/nop2.1470 PMC10006579

[52] Gustavson K , Knudsen AK , Nesvåg R , Knudsen GP , Vollset SE , Reichborn‐Kjennerud T . Prevalence and stability of mental disorders among young adults: findings from a longitudinal study. BMC Psychiatry. 2018;18(1):65. 29530018 10.1186/s12888-018-1647-5 PMC5848432

[53] Mojtabai R , Olfson M , Han B . National trends in the prevalence and treatment of depression in adolescents and young adults. Pediatrics. 2016;138(6):e20161878. 27940701 10.1542/peds.2016-1878 PMC5127071

[54] Maslow AH . A theory of human motivation. Psychol Rev. 1943;50(4):370–396.

[55] Kwon C , Han S , Nicolaides A . The impact of psychological safety on transformative learning in the workplace: a quantitative study. J Workplace Learn. 2020;32(7):533–547.

[56] Newman A , Donohue R , Eva N . Psychological safety: a systematic review of the literature. Hum Resour Manag Rev. 2017;27(3):521–535.

[57] Hallam KT , Popovic N , Karimi L . Identifying the key elements of psychologically safe workplaces in healthcare settings. Brain Sci. 2023;13(10):1450. 37891818 10.3390/brainsci13101450 PMC10605501

[58] Valizadeh L , Zamanzadeh V , Gargari RB , Ghahramanian A , Tabrizi FJ , Keogh B . Pressure and protective factors influencing nursing students’ self‐esteem: a content analysis study. Nurse Educ Today. 2016;36:468–472. 26586259 10.1016/j.nedt.2015.10.019

[59] Cheung KL , ten Klooster PM , Smit C , de Vries H , Pieterse ME . The impact of non‐response bias due to sampling in public health studies: a comparison of voluntary versus mandatory recruitment in a Dutch national survey on adolescent health. BMC Public Health. 2017;17(1):276. 28330465 10.1186/s12889-017-4189-8 PMC5363011

[60] Bahous SA , Salameh P , Salloum A , Salameh W , Park YS , Tekian A . Voluntary vs. compulsory student evaluation of clerkships: effect on validity and potential bias. BMC Med Educ. 2018;18(1):9. 29304800 10.1186/s12909-017-1116-8 PMC5756350

